# Aberrant Expression of a Proliferation-Inducing Ligand Underlies Autoimmune Mechanisms in Immune Thrombocytopenia

**DOI:** 10.1155/2021/3676942

**Published:** 2021-01-30

**Authors:** Y. F. Hao, H. Bi, H. Y. Li, L. M. Yin, J. X. Yu, W. Tao, H. L. Mu, R. C. Yang, Z. P. Zhou, W. L. Tai

**Affiliations:** ^1^Department of Hematology, Second Affiliated Hospital of Kunming Medical University, Yunnan, Kunming 650101, China; ^2^State Key Laboratory of Experimental Hematology, Institute of Hematology and Blood Disease Hospital, Chinese Academy of Medical Sciences & Peking Union Medical College, Tianjin 300020, China; ^3^Yunnan Molecular Diagnosis Center, Second Affiliated Hospital of Kunming Medical University, Yunnan, Kunming 650101, China

## Abstract

**Purpose:**

To study the relationship between surface membrane-bound APRIL and ITP.

**Methods:**

The peripheral blood of all subjects, 50 patients diagnosed with ITP and 25 healthy controls, was collected. Flow cytometry was used to detect the expression of membrane-bound APRIL on immune cells and platelets. ELISA was used to detect the content of soluble APRIL in plasma.

**Results:**

Membrane-bound APRIL was only expressed on the surface of platelets in both ITP patients and controls. APRIL expression on the platelet surface was significantly lower in newly diagnosed (*P* < 0.001) and chronic (*P* < 0.001) ITP patients than in controls. Platelet surface APRIL level was significantly enhanced in patients with complete remission after treatment (*P* = 0.02) but not in those with no response after treatment. Platelet surface APRIL level in ITP patients was negatively correlated with serum APRIL level (*r* = −0.09765, *P* = 0.0424).

**Conclusions:**

Platelet surface APRIL may play a key immunoregulative role. Platelet surface APRIL is likely to be one source of the excessive serum APRIL in ITP patients. The effectiveness of treatment may be measured by determining the platelet surface APRIL levels in ITP patients.

## 1. Introduction

Primary immune thrombocytopenia (ITP) is an antibody-mediated autoimmune disease characterized by accelerated platelet destruction and aberrant platelet production. While it may be asymptomatic for some patients, others can suffer mild, moderate, or even severe bleeding. The pathophysiology of ITP is complex but may be caused by multiple factors such as impaired megakaryopoiesis leading to reduced platelets [1], the proliferation of autoreactive T cells particularly the upregulation of Th17 and downregulation of Tregs [2], increased antiplatelet autoantibodies by B lymphocytes [3], and loss of peripheral immune tolerance [4].

APRIL, also known as tumor necrosis factor superfamily [TNFSF] 13, is secreted by a range of cells including myelocytes [5], activated platelets [6], and activated T and B cells [5]. Structurally and functionally similar to B cell-activating factor (BAFF), APRIL is believed to play a pivotal immunoregulatory role in patients with various autoimmune diseases including [7–9]. Increased levels of APRIL have been found in different autoimmune diseases such as systemic lupus erythematosus [10], rheumatoid arthritis (RA) [11], Sjogren's syndrome (SS) [12], and ITP [13, 14]. Previous studies have shown that the pathogenesis of ITP may be related to an increased level of a proliferation-inducing ligand. In ITP patients, the serum level of APRIL cannot be lowered by glucocorticoid therapy which can significantly reduce BAFF [15, 16], but may be effectively reduced by vitamin D3 [15]. Consensus has not been reached as to the origin of the increase in serum APRIL level. Gu et al. proposed that APRIL originates from increased gene expression. By studying the predisposing gene in familial or sporadic ITP, Peng et al. show that the p.G76S mutation on the TNFRSF13B gene in ITP patients may lead to enhanced binding ability of APRIL ligands to B cells as well as increased BAFF levels [17]. In contrast, Kamhieh-Milz et al. conclude that APRIL has nothing to do with increased gene expression [15].

Since APRIL is found to contribute to platelet production as an autocrine growth factor produced by megakaryocytes [18], its role for ITP patients can be essential. However, studies on the relationship between APRIL and ITP are few and far between. Of the few published articles on the topic, three are particularly relevant to the current paper. The first is Gu et al. which suggests that plasma APRIL level in ITP patients was significantly higher than that in healthy controls and correlated closely with platelet count [14]. Gu and his colleagues also attempted to understand the reason of the increased APRIL in the plasma of ITP patients by performing real-time quantitative polymerase chain reaction to determine the mRNA expression of APRIL. Based on the result of mRNA expression of APRIL by PBMNCs, they speculated that PBMNCs may be a source of the excessive APRIL. While Gu and his colleagues have shed important light on the role of APRIL for ITP patients, more research is needed on this topic for three reasons.

Firstly, they overlooked surface membrane-bound APRIL, since they only examined soluble APRIL in plasma. There are two forms of APRIL: soluble and surface membrane-bound [19]. It is believed that surface membrane-bound APRIL is hardly detectable due to its extremely low expression [19]. This is likely the reason that these researchers focused only on soluble APRIL. However, more recent studies detected surface APRIL in patients suffering from diseases such as leukemia and RA [20–22]. Therefore, researchers interested in the relationship between APRIL and ITP need to take surface membrane-bound APRIL into consideration. Secondly, the method used by Gu and his colleagues to explore the reason behind increased APRIL in the plasma of ITP patients, real-time quantitative polymerase chain, seems somewhat circuitous. It may be more straightforward to detect that by flow cytometry. Thirdly, they did not compare the changes in APRIL levels before and after treatment in ITP patients. This information is vital because it shows disease activity in relation to changes in APRIL.

Kamhieh-Milz et al. [15], another study on the relationship between APRIL and ITP, compared serum levels of BAFF and APRIL in active ITP patients before and after treatment with GC. The former decreased significantly by glucocorticoid treatment alone, while the latter only responded well after adding vitamin D3 to prednisolone. While GC has remained the primary therapy for ITP patients, it is frequently used in combination with other medications such as DMN, IVIg. In some cases, doctors even give up GC due to uncontrollable bleeding. Probably, for this reason, Álvarez Román et al., a third study relevant to the current paper, investigated the effect of TPO on the level of plasma APRIL in ITP patients and revealed that this medication significantly reduced plasma APRIL level [21]. However, it remains questionable whether the findings of Kamhieh-Milz et al. and Butta et al. can be readily generalized to patients who receive medications other than treatment in their studies.

In this study, we examine the relationship between APRIL and ITP by focusing on surface membrane-bound APRIL with two objectives in mind: (1) to find out whether APRIL is expressed on the surface of immune cells and platelets in ITP patients and normal controls; (2) if yes, how surface membrane-bound APRIL respond to treatment; and (3) to explore how surface membrane-bound APRIL is related to soluble APRIL.

## 2. Materials and Methods

### 2.1. Participants

50 ITP patients and 25 healthy controls participated in the study. The ITP patients (29 females, 21 males; mean age: 47.5 years, range: 24–78 years) visited the Institute of Hematology, Chinese Academy of Medical Sciences Hematology Hospital from July 2017 to June 2018. ITP was diagnosed based on recently reported standards. Among the 50 patients, 25 were newly diagnosed (17 women and 8 men; median age: 45 years, range: 24–76 years) with a median platelet count of 19 × 109/l (range: 1–44 × 109/l) at diagnosis, and the other 25 were chronic ITP patients (>1 year after diagnosis; 13 women and 12 men; median age: 51 years, range: 25–78 years) with a median platelet count of 44 × 109/l at diagnosis (range: 3–165 × 109/l).

Treatment response was determined based on a consensus of Chinese haematologists [23]. That is, complete response (CR) was defined as a platelet count ≥100 × 109/l and absence of bleeding; no response (NR) was rated as platelet count <30 × 109/L or less than 2-fold increase of baseline platelet count or bleeding.

The control group comprised of healthy, age-matched subjects. The baseline characteristics of the patients and controls are summarized in Table 1.

### 2.2. Preparation of Platelet Suspension

The EDTA blood was centrifuged at 150 × g for 10 minutes to obtain platelet-rich plasma samples, which were then transferred to polypropylene test tubes. PRP was centrifuged at 2,300 × g for 5 minutes to obtain platelet pellets, which were then resuspended in ammonium oxalate (1%) for erythrocyte lysis. After centrifuging at 1100 × g for 5 minutes to discard the supernatant, the pellet was washed twice in phosphate-buffered saline (PBS) containing EDTA (0.009 mol/l Na2EDTA,0.0264 mol/l Na2HPO4, 0.07 mol/l NaCl). The concentration of platelets in PBS-EDTA buffer was 1 × 10^7^/ml [24–26].

### 2.3. Flow Cytometry Analysis

The platelets were stained with Anti-CD41-FITC (San Diego, CA, USA), Anti-CD61-APC (San Diego, CA, USA), and Anti-APRIL-PE (R&D Systems) at room temperature for 30 minutes. The platelets were then washed twice with PBS-EDTA buffer and resuspended in 0.5 ml of PBS-EDTA buffer and immediately analyzed on a flow cytometer. The platelets were sorted on a FACS Canto II instrument (BD Biosciences, Franklin Lakes, NJ, USA), and data were analyzed with the FlowJo v.10.0 software (Tree Star, Ashland, OR, USA).

Peripheral blood was collected into EDTA-anticoagulant vacuum tubes. PBMCs were isolated using lymphoprep density gradient centrifugation (Haoyang, Tianjin, China). PBMCs cells were stained with various combinations of monoclonal antibody (mAb) for 30 min on ice in staining buffer (1% BSA in PBS). The directly conjugated mAbs used were anti-CD19-FITC (BioLegend), anti-CD3-APC (BioLegend), anti-CD14-PECY7 (BioLegend) and anti-APRIL-PE (R&D Systems). Stained cells were washed twice with 2 ml PBS buffer, fixed in 1% paraformaldehyde, and analyzed within 24 h. The data were analyzed with the FlowJo v.10.0 software (Tree Star, Ashland, OR, USA).

### 2.4. Determination of Plasma APRIL Levels

Peripheral blood was collected into EDTA-anticoagulant vacuum tubes. Plasma was separated from blood cells by centrifugation (2600 g, 5 min) and was frozen at −80°C until analysis. PBMCs were isolated using lymphoprep density gradient centrifugation (Haoyang, Tianjin, China). APRIL levels in patient and control plasma were detected by antigen capture enzyme-linked immunosorbent assay using a commercial kit (BioLegend) according to the manufacturer's instructions. Plasma APRIL concentration was calculated from a standard curve generated using purified human APRIL supplied with the kit.

### 2.5. Statistical Analysis

Data are expressed as mean ± standard error of the mean and were analyzed using SPSS v.13.5 (SPSS Inc., Chicago, IL, USA). Factor variance analysis or *t*-test was used for comparison of sample mean within the group. The Pearson correlation coefficient or Spearman rank correlation was used to analyze the correlation between variables. The results before and after treatment were analyzed by paired *t*-test. *P* < 0.05 was considered statistically significant. All experimental analyses were performed using the GraphPad Prism 7.0 software.

## 3. Results

The APRIL level of both CR and NR ITP patients is shown in Table 2. Below, we elaborate the major findings.

### 3.1. APRIL Was Only Expressed on the Surface of Platelet

**Figure 1:**
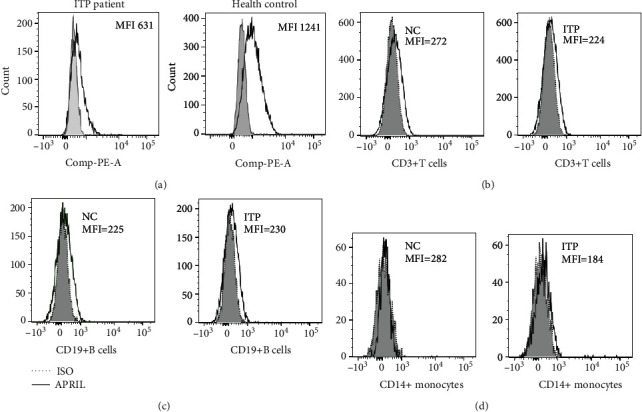
Representative histograms show surface APRIL and isoform staining of platelets (a), B cells (b), T cells (c), and monocytes (d).

Compared with isotype controls, surface APRIL could be clearly detected only in platelets in all ITP patients and normal controls. The mean fluorescence intensity (MFI) of platelet surface APRIL staining in ITP patients and normal controls was 631 and 1241, respectively. In contrast, little surface APRIL was observed in other immune cells. Specifically, the MFI of APRIL on the surface of CD19 + B cells in ITP patients and normal controls was 230 and 225, on that of CD3 + T cells being 224 and 272 and on that of CD14 + monocytes being 184 and 282, respectively.  Figure 1 shows surface APRIL and isoform staining of platelets (Figure 1(a)), B cells (Figure 1(b)), T cells (Figure 1(c)), and monocytes (Figure 1(d)).

### 3.2. Platelet Surface APRIL Expression Was Downregulated in ITP Patients

**Figure 2:**
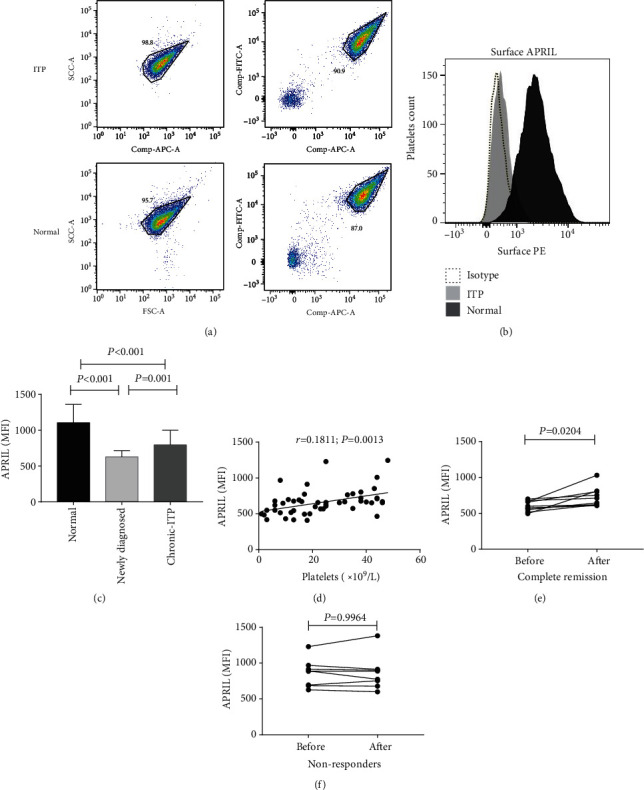
Decreased surface expression of APRIL on platelets in ITP patients. (a) Platelets from healthy donors and ITP patients were isolated by flow cytometry based on surface expression of APRIL. Forward and side light scatter (FSC and SSC, respectively) and intact cell gates (left panels) were used for platelet gating (right panels). (b) Representative histograms show surface APRIL and isotype labeling in gated platelets. (c) MFI of surface APRIL expression in gated platelets from healthy donors and ITP patients. (d) Spearman's test showed that APRIL expression in ITP patients (*n* = 50) was highly correlated with platelet count. (e, f) Differences in APRIL MFI in ITP patients in complete remission (*n* = 10) (e) and nonresponders (*n* = 8) (f) before and after treatment.

Compared with isotype controls, surface APRIL can be clearly detected on the surface of gated platelets in all ITP patients and normal controls (Figure 2(b)). A graph comparing APRIL stained MFI on platelet surfaces from the 25 healthy controls and 50 ITP patients is provided (Figure 2(c)). APRIL expression on the platelet surface was significantly reduced in newly diagnosed (*P* < 0.001) and chronic (*P* < 0.001) ITP patients relative to controls.

Correlation analysis of the MFI of the APRIL on platelet surface of ITP patients and the platelet count suggests that they were positively correlated with each other (*r* = 0.1811, *P* = 0.0013) (Figure 2(d)).

### 3.3. Effective Treatment Enhanced Platelet Surface Level

The changes of platelet surface APRIL were monitored before and after treatment in active ITP patients (*n* = 18). In patients with complete remission, APRIL MFI on platelet was higher after treatment than before treatment in the CR group (721.3 ± 42.39 vs. 599.5 ± 22.28, *n* = 10, *P* = 0.0204) (Figure 2(e)), whereas little difference was observed in the NR patients (861.3 ± 68.79 vs. 860.8 ± 84.17, *n* = 8, *P* = 0.9964) (Figure 2(f)).

### 3.4. Plasma APRIL Level Was Elevated in ITP Patients Regardless of Treatment

**Figure 3:**
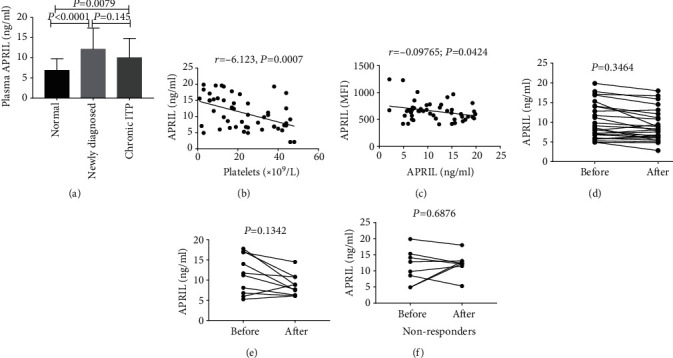
Plasma APRIL levels in ITP patients. (a) Plasma APRIL levels were higher in chronic and newly diagnosed ITP patients than in normal controls (*n* = 25 per group), with no significant difference between the two patient groups. (b) APRIL concentration was negatively correlated with platelet count in ITP patients (*n* = 50). (c) Correlation between platelet surface APRIL and serum APRIL levels in ITP patients. (d–f) Differences in Plasma APRIL levels before and after treatment in the chronic ITP patient group (*n* = 25) (d) in the complete remission group (*n* = 10) (e) and the nonresponse group (*n* = 8) (f).

The average serum APRIL concentration in the normal control group was 6.87 ± 0.58 ng/ml (range: 5.67–8.07 ng/ml) (Figure 3(a)). The mean concentration in newly diagnosed ITP patients was 12.1 ± 1.04 ng/ml (range: 9.94–14.27 ng/ml, *P* < 0.001) and that in chronic ITP patients was 10.0 ± 0.96 ng/ml (range: 8.01–11.98 ng/ml, *P* = 0.0079). These values were significantly higher than that of the normal control group.

Serum APRIL levels were negatively correlated with platelet counts in ITP patients (*r* = −6.123, *P* = 0.0007) (Figure 3(b)). Platelet surface APRIL in ITP patients was negatively correlated with serum APRIL level (*r* = −0.09765, *P* = 0.0424) (Figure 3(c)). After treatment, there was no significant difference in plasma APRIL levels in ITP patients (10.2 ± 0.9144 vs. 9.036 ± 0.808, *n* = 25, *P* = 0.3464) (Figure 3(d)). There was no significant change in plasma APRIL before and after treatment in ITP patients in the CR group (11.49 ± 1.528 vs. 8.75 ± 0.8446, *n* = 10, *P* = 0.1342) and the NR group (11.28 ± 1.846 vs. 12.19 ± 1.215, *n* = 8, *P* = 0.6876) (Figures 3(e) and, 3(f)).

## 4. Discussion

In this study, we investigated the relationship between APRIL and ITP, generating some interesting results. We began with exploring whether and how APRIL was expressed on the surface of immune cells and platelets in ITP patients and normal controls. We found that APRIL was saliently expressed only on the surface of platelets in both ITP patients and normal controls. Compared with controls, platelet surface APRIL was significantly reduced in ITP patients. This trend was obviously reversed in ITP patients with complete remission after treatment. In contrast, for patients who did not respond to treatment, the platelet surface APRIL level remained similar before and after treatment. We interpret our findings below.

We were triggered by recent studies that detected surface APRIL in patients who suffered from diseases such as leukemia and RA [20–22] to ask whether membrane-bound ARPIL expresses on the cell surface of ITP patients. To answer this question, we measured the expression of APRIL on the surface of platelets and peripheral mononuclear cells including T cells, B cells, monocytes, and dendritic cells from ITP patients and normal controls by flow cytometry. We found that membrane-bound APRIL was only expressed on the surface of platelets in both ITP patients and controls. Meanwhile, surface platelet APRIL in patients remained significantly lower than that in normal controls. Thus, we join in Weldon et al. to challenge the proposition that APRIL was hardly expressed on cell surface [18, 19]. In the study of Weldon and his colleagues, the surface form of APRIL has been identified in lymphoid and bone marrow mononuclear cells and macrophages in synovial tissue of RA patients [22, 27]. They explained the mechanism for cell surface APRIL expression by referring to alternative APRIL splice forms and TWE-PRIL [28]. While that study revealed that RA patients manifested higher levels of surface form of APRIL than normal controls, ITP patients in this study showed a lower level of platelet surface APRIL than normal controls. We speculate that this contrast may result from the difference in disease types.

Our finding that the platelet surface APRIL level in the ITP patients fell significantly below the controls may be explained by five reasons. Firstly, it may be related to the imbalance of furin, since APRIL is processed intracellularly by furin convertase. While research on ITP on this issue is lacking, it has been reported that furin protease dysregulation is associated with increased levels of BAFF and APRIL in immune cells of patients with advanced atherosclerotic plaque [29]. The second possible reason is that some surface platelet APRIL shed into the plasma due to unknown pathophysiological reasons [14, 17]. If this reason can be confirmed in the future, we can say that surface platelet APRIL is another source of soluble APRIL increase in plasma in ITP patients. The third possible reason is that megakaryocytes can transfer MHC class I molecules loaded with foreign antigen to proplatelets in vitro [30]. The fourth possible reason for the decrease of surface APRIL in the platelet of ITP patients is the preactivation of platelet, which can significantly reduce the expression of platelet surface markers with little change in platelet function [31].

An additional explanation for the plummet of platelet surface APRIL level in the ITP patients may be related to ADAM17 [32]. It has been reported that metalloproteinases ADAM17 can trigger the combination of BAFFR and TACI. Inhibition of ADAM17 can increase the expression level of BAFFR on germinal center B cells. In a similar vein, the inhibition of ADAM17 may also increase the expression level of APRIL in plasma. ADAM17 stimulates the shedding of platelet receptor (e.g., platelet-derived growth factor receptor *β*), TNF and its receptors (TNFR-1 and TNFR-2), and Glycoprotein V [33–35]. Since the mechanism of the cleavage effect of ADAM17 on APRIL remains unclear, further research is desirable.

Next, we explored how treatment affected the expression of surface APRIL in ITP patients. We found that, for CR patients, the platelet surface APRIL level after treatment was significantly higher than that before treatment. In contrast, for NR patients, platelet surface APRIL level remained similar before and after treatment. This finding seems to suggest that surface APRIL may also play a role in immunoregulation together with soluble APRIL, again challenging Lopez-Fraga et al. who suggested that only soluble APRIL has an immunoregulation role [18]. At present, the exact mechanism for the role of surface APRIL in immunoregulation remains unclear. Regardless of this, our finding may be clinically significant as the effectiveness of treatment may be measured by determining surface APRIL levels in ITP patients.

To sum up, membrane-bound APRIL was only expressed on the surface of platelets in both ITP patients and normal controls with platelet surface APRIL in the former significantly lower than that in the latter. Platelet surface APRIL was reversed by treatment for CR patients but not for NR ones. Platelet surface APRIL may be a source of the excessive APRIL in the plasma of ITP patients. Aberrant APRIL expression on the surface of platelets is likely linked to autoimmune response in ITP patients. Our findings not only suggest important future research directions but also are clinically significant.

## Figures and Tables

**Table 1:** Basic characteristics of ITP patients.

Patients	Gender	Age (year)	Platelet	Therapy	Patients	Gender	Age (year)	Platelet	Therapy	Controls	Gender	Age (year)
1	M	33	3	—	26	F	28	17	GC	1	M	22
2	F	24	24	—	27	F	22	8	GC	2	M	29
3	F	43	13	—	28	F	63	13	GC	3	F	35
4	F	64	19	—	29	F	34	38	GC, TPO	4	M	45
5	F	51	12	—	30	M	54	32	GC	5	F	59
6	F	61	33	—	31	M	58	107	GC	6	F	58
7	F	57	38	—	32	M	31	42	DNZ	7	M	44
8	F	43	34	—	33	F	40	121	GC, DNZ	8	M	42
9	M	48	9	—	34	F	51	18	DNZ	9	M	28
10	F	54	25	—	35	F	68	34	GC	10	F	20
11	M	76	22	—	36	M	45	160	GC	11	M	62
12	F	51	16	—	37	F	69	25	GC, DNZ	12	F	34
13	F	28	1	—	38	F	26	109	GC, DNZ	13	F	27
14	M	38	6	—	39	F	53	17	GC	14	F	36
15	M	45	6	—	40	F	26	123	GC	15	F	45
16	M	48	2	—	41	F	35	3	GC	16	M	46
17	M	63	25	—	42	M	23	35	GC, IVIg	17	F	51
18	F	54	44	—	43	M	46	113	GC, IVIg	18	F	22
19	F	27	35	—	44	M	22	24	GC	19	F	35
20	F	44	46	—	45	M	50	116	GC, DNZ	20	M	37
21	F	35	13	—	46	M	74	48	GC	21	M	40
22	F	28	18	—	47	M	50	38	GC	22	F	63
23	F	36	40	—	48	M	53	46	GC	23	F	65
24	M	33	43	—	49	M	27	3	GC	24	M	33
25	F	63	10	—	50	M	40	15	GC	25	M	28

M: male; F: female; GC: hormone; TPO: thrombopoietin; DNZ: danazol; IVIg: intravenous immunoglobulin.

**Table 2:** Basic characteristics of chronic ITP patients.

Patients	Group	Gender	Age (year)	Platelet	Before treatment APRIL level (ng/ml)	After treatment APRIL level (ng/ml)	Before treatment APRIL MFI	After treatment APRIL MFI
26	CR	F	28	17-265	17.8	8.8	678	495
27	NR	F	22	8-19	15.3	12.3	710	968
28	NR	F	63	13-16	9.8	11.5	668	678
29	CR	F	34	38-143	6.8	6.1	619	678
30	R	M	54	32-88	5.9	6.0	654	668
31	CR	M	58	8-207	11.7	10.8	618	515
32	R	M	31	42-90	5.7	6.6	913	654
33	CR	F	40	12-132	16.9	14.5	1009	599
34	NR	F	51	18-21	14.09	12.1	803	913
35	R	F	68	34-89	7.9	9.4	1381	1009
36	CR	M	45	30-160	8.1	6.3	1245	655
37	NR	F	69	25-29	4.9	12.3	890	1229
38	CR	F	26	25-109	14.03	7.5	646	659
39	NR	F	53	17-23	12.8	13.1	627	774
40	CR	F	26	6-123	17.12	10.8	607	550
41	NR	F	35	3-17	19.86	17.98	753	599
42	CR	M	23	35-294	11.2	7.7	711	576
43	CR	M	46	11-113	5.97	8.9	617	699
44	R	M	22	24-44	7.2	7.5	753	711
45	CR	M	50	23-116	5.26	6.1	721	569
46	R	M	74	48-80	7.09	8.1	655	1245
47	R	M	50	38-87	6.9	6.7	678	721
48	R	M	53	46-90	9.1	8.3	710	655
49	NR	M	27	3-13	4.91	12.9	668	885
50	NR	M	40	15-25	8.57	5.32	619	694

M: male; F: female; CR: complete response; R: response; NR: no response.

## Data Availability

The statistical analysis (as Excel and Statistica files) used to support the findings of this study are available from the corresponding author upon request. email(zhouzeping@kmmu.edu.cnz).
